# Editorial: Advances in ripening regulation, quality formation, pre and post-harvest applications of horticultural products

**DOI:** 10.3389/fpls.2023.1285104

**Published:** 2023-09-14

**Authors:** Yudong Liu, Guojian Hu, Mondher Bouzayen, Zhengguo Li

**Affiliations:** ^1^ National Horticulture Germplasm Resources Center, School of Agricultural Sciences, Zhengzhou University, Zhengzhou, China; ^2^ Université de Toulouse, INRAe/INP Toulouse, Génomique et Biotechnologie des Fruits—UMR990, Castanet-Tolosan, France; ^3^ Key Laboratory of Plant Hormones and Development Regulation of Chongqing, School of Life Sciences, Chongqing University, Chongqing, China

**Keywords:** fruit, ripening, quality, phytohormone, transcription factor

Horticultural crop products, such as fruits, provide important health-supporting nutrients in human diet. The quality of fruits is closely related to consumer-liking, influences fruit consumption and the development of fruit industry. Fruit ripening is a complex process with dramatic changes in color, texture, flavor, aroma, and nutrient metabolism that determines fruit quality formation. Fruit ripening is regulated by a number of transcription factors (TFs) in conjunction with the plant hormones, epigenetic modifications, as well as exogenous environment factors. Therefore, efforts to decipher the mechanisms of ripening regulation and quality formation are crucial contributing novel strategies to improve fruit quality. This Research Topic contains 4 original research articles that shed new light on the regulation mechanism of fruit ripening and quality formation or provide effective strategies to improve fruit quality.

Yi et al. identified an ARF family transcription factor FveARF2, which is located in the nucleus, and plays as a negative regulator of strawberry fruit ripening and quality. By both silencing and overexpression of the *FveARF2* gene in strawberry, the authors demonstrated that *FveARF2* negatively regulates fruit earlier coloration, content of soluble solid, sugar, and anthocyanin through directly targeting genes associated with fruit quality formation, such as *FaSUT1*, *FaOMT*, and *FaCHS*. In addition, the authors also proposed that *FveARF2* negatively regulate strawberry fruit quality by affecting potassium ion transport through directly binding to promoter of a potassium transporter gene *FveKT12*. The expression of *FveARF2* is negatively correlated with content of potassium ion, and silencing *FveARF2* increased potassium ion content in transgenic fruit. This study provides a new candidate gene resource for controlling strawberry fruit ripening and quality through molecular breeding.

Post-harvest ripening is a crucial course for banana fruit with significant conversion of starch. Xiao et al. screened the potential upstream regulators of *MaGWD1*, a key gene related to starch degradation of post-harvest banana fruit, and identified an ERF-AP2 family transcription factor, MaAP2a, could directly targets the promoter of *MaGWD1*. MaAP2a encompasses a transcriptional repressor domain (EAR, LxLxLx) with transcriptional inhibitory activity, and is located in the nucleus. Protein-DNA interaction assays such as electrophoretic mobility shift assay, Chromatin immunoprecipitation-qPCR analysis, and Dual-luciferase reporter assay demonstrated that MaAP2a directly binds to the promoters (GCC-box or AT-rich motif) of 15 starch degradation-related genes *MaGWD1*, *MaPWD1*, *MaSEX4*, *MaLSF1*, *MaBAM1/2/3*, *MaAMY2B/2C/3A/3C*, *MaMEX1/2*, and *MapGlcT2-1/2-2*, and repressed their activity. The authors confirmed a transcriptional repressor MaAP2a that participated in post-harvest banana ripening with a negative influence on starch degradation by directly targeting a series of starch degradation-related genes. This study shed light on the control of starch degradation of banana fruits during post-harvest ripening.

Phytohormones, such as ethylene (ET), gibberellin (GA), auxin and the interaction of them play important roles in fruit ripening and quality formation. Park and Malka performed transcriptome and metabolomic analysis of tomato fruit treated with ET, GA, and ET+GA to identify the potential molecular mechanism of ET and GA interplay in tomato fruit ripening. The authors found that ET and GA play opposite effects on auxin signaling and metabolite changes (such as sugar, amino acids, organic acids), and GA alone or combined with ET could inhibit ET signaling. This research indicated that auxin is involved in the interplay between ET and GA, enriched our understanding of the interaction of phytohormones during fruit ripening.

Li et al. evaluated the effects of alginate oligosaccharides (AOS) application on the antioxidant system, photosynthesis and fruit sugar accumulation in citrus through a two-year field experiment. The authors determined the optimal spray concentration and cycle of AOS on pre-harvest citrus fruit that could increase the content of soluble sugars and soluble solids. And the authors confirmed that the increase of sugar content in citrus fruits with AOS treatment may be related to the increased antioxidant enzyme activity and photosynthetic rate in leaves, as well as the transport of leaf assimilation product. Overall, the authors provide a potential application of AOS in the production of sugar enriched citrus fruits.

The articles in this Research Topic provide advanced information in fruit ripening regulation, quality formation, and pre-harvest applications. Further studies of endogenous and exogenous factors, such as phytohormones, TFs, epigenetic modifications, as well as environment factors that are involved in horticulture crops ripening and quality formation will be helpful to improve crop quality.

## Author contributions

YL: Writing – review & editing, Conceptualization, Project administration, Validation, Writing – original draft. GH: Writing – review & editing, Conceptualization, Project administration, Validation, Writing – original draft. MB: Conceptualization, Project administration, Validation, Writing – original draft, Writing – review & editing. ZL: Writing – review & editing.

